# Midterm Outcomes of Crosslinked Acellular Bovine Jugular Vein Conduit for Right Ventricular Outflow Tract Reconstruction

**DOI:** 10.3389/fped.2021.725030

**Published:** 2021-08-17

**Authors:** Tao Qian, Zhong-Shi Wu, Jian-Guo Hu, Yi-Feng Yang, Qin Wu, Ting Lu, Can Huang, Jia Li

**Affiliations:** ^1^Department of Cardiovascular Surgery, The Second Xiangya Hospital, Central South University, Changsha, China; ^2^Engineering Laboratory of Hunan Province for Cardiovascular Biomaterials, Changsha, China; ^3^Clinical Physiology Laboratory, Guangzhou Women and Children's Medical Center, Institute of Pediatrics, Guangzhou Medical University, Guangzhou, China; ^4^Guangdong Provincial Key Laboratory of Research in Structural Birth Defect Disease, Guangzhou Women and Children's Medical Center, Guangzhou Medical University, Guangzhou, China

**Keywords:** bovine jugular vein conduit, decellularized and photooxidatively crosslink, right ventricular outflow tract reconstruction, congenital heart disease, outcomes

## Abstract

**Objectives:** Conduits for reconstructing right ventricular outflow tract (RVOT) in children with congenital heart disease have evolved for better durability over the past decades, but conduits failure remains common. We designed decellularized and photooxidatively crosslinked bovine jugular vein conduit (DP-BJVC) and now aim to evaluate the midterm results of DP-BJVC for RVOT reconstruction.

**Methods:** Ninety patients (median age: 4.2 years) undergoing RVOT reconstruction using DP-BJVC were prospectively followed for median of 4.7 years (range: 0.2–16.1 years). Kaplan–Meier analysis was used to examine the survival, freedom from conduit explantation and catheter-based reintervention. Risk factors were analyzed with Cox regression analysis.

**Results:** Follow-up was completed in 92% of patients. There were five (5.6%) early deaths. The 10-year survival rate was 85.2%, with palliative procedure at DP-BJVC implantation as the risk factor. The 10-year freedom from conduit explantation and reintervention were 84.4 and 67.3% respectively, with previous cardiac operation as the only risk factor for explantation. Complications during the follow-up included conduit stenosis (peak gradient ≥50 mmHg) in 12 (12.9%), severe regurgitation in 2 (2.4%), and infective endocarditis in 2 (2.4%). The annual increase in gradient was highest in the first year (*P* = 0.003), but not appreciably afterwards. The echo-measured annulus diameter trends to increase by an average of 0.37 mm per year. Calcification appeared mild in the failed conduits.

**Conclusions:** DP-BJVC provides satisfactory durability and functionality for RVOT reconstruction for children, with low morbidity of stenosis and endocarditis, as well as increase in diameter mildly with age in midterm follow-ups.

**Graphical Abstract:**
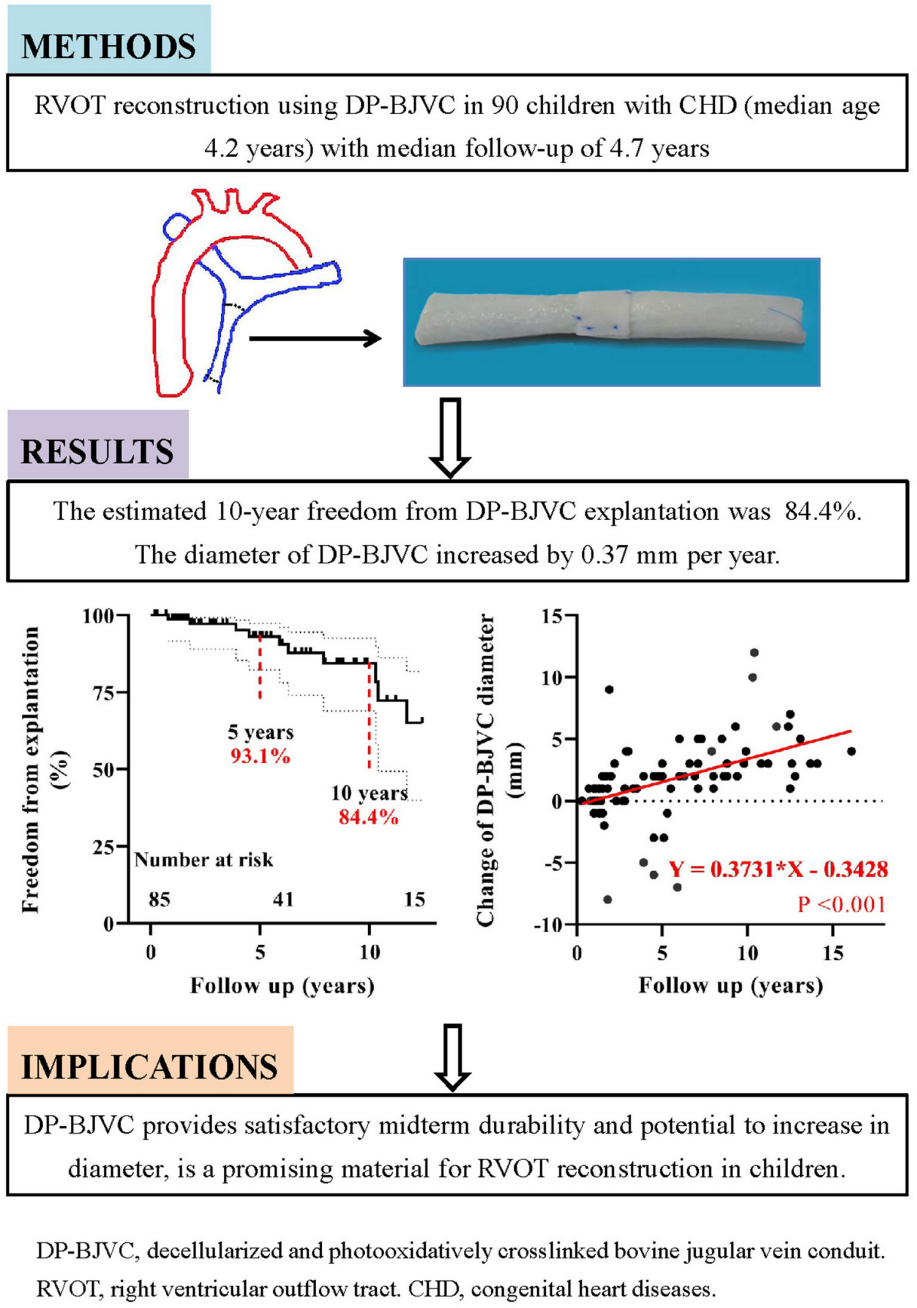
Midterm performance of DP-BJVC for RVOT reconstruction in 90 patients with median follow-up of 4.7 years showed good durability and the potential to increase in diameter.

## Introduction

The surgical reconstruction of right ventricular outflow tract (RVOT) with valved conduit remains a common cardiac procedure for patients with congenital heart diseases (CHD) (1). However, explantation remains common for the currently available conduits, thus improving the durability of RVOT conduits in young children remains a challenge. Younger age at implantation was the leading adverse factor of the valved conduits failure (2–4), mainly as the result of fast somatic growth. The durability of RVOT conduit in young children remains to be improved yet challenging.

Historically, cryopreserved pulmonary homograft was the “gold standard” from the mid-1980s through the late 1990s. Due to concerns of limited durability, availability and costs of homografts, attention has turned to xenograft and artificial material as alternatives. The Contegra conduit (Medtronic Inc., Minneapolis, Minnesota, USA), a glutaraldehyde-treated bovine jugular vein conduit (BJVC) developed in 1990s, is one of the most successful alternates. It makes up the shortage of homograft and costs less. Its hemodynamic performance and durability have been found comparable to homograft (5–8). Nonetheless, <25% of infants receiving the glutaraldehyde-treated BJVC were free from replacement at 10 years (4, 5, 9). The glutaraldehyde brings unpredictable cytotoxicity and possibility of early calcification (10). Moreover, the graft-related immune response induced by residual donor cells and cell debris plays an important role in the failure of glutaraldehyde-treated xenografts (11). The latter factor may be avoided by decellularization, which has been adopted in cardiovascular biomaterials over 30 years (12). The decellularized homografts have significantly improved functionality compared with standard cryopreserved homograft used for RVOT reconstruction (13). Recently, the Japanese approach of hand-sewn expanded polytetrafluoroethylene (ePTFE) valved conduit has attracted attention, with satisfactory long-term outcomes from Japanese multi-center studies (14). But its application was limited largely within Japan by the inconsistent quality control of hand-sewn valve until recent years.

Our team started working on the non-glutaraldehyde-treatment of BJVC combined with decellularization and photooxidatively crosslinking (DP-BJVC) since 2002 (15). We hypothesized that DP-BJVC prevents the immunogenicity from xenogeneic cells and avoids the toxic effects of glutaraldehyde. In a series of animal experiments in rats or dogs, DP-BJVC was shown to have favorable biocompatibility and tissue structure stability, and greater calcification resistance than glutaraldehyde-treated BJVC (16, 17). In the past 16 years, DP-BJVC has been used clinically in our center. The aim of this study was to evaluate the outcomes of our cohort.

## Patients and Methods

### Patients

After approved by the Institutional Ethics Committee (reference number: LYF2004011) and written informed consent obtained from patients or their guardians, 122 patients were prospectively enrolled for DP-BJVC implantation. The study protocol was also approved (reference number: LYF2020097). Among 122 patients, 32 were excluded from this study. Two patients underwent superior vena cava reconstruction using DP-BJVC, and 30 received DP-BJVC implantation in the form of valved patch.

### DP-BJVC Preparation

The conduit was prepared with the multi-step detergent-enzymatic decellularization and dye-mediated photooxidation procedures as previously reported (16, 17). The DP-BJVC (Yaxin Medical Technology Co., Ltd., Wuhan, Hubei, China.) consists of natural tri-leaflet valve with size ranging from 12 to 20 mm with 1 mm as spacing. The annulus restraining device located at the level of valve annulus mainly fixed on the outer wall of the conduit, was made to enhance the conduit stability since 2013. The device was a rectangular Dacron patch wrapped loosely around the conduit, and gently held together circumferentially by several 6-0 Prolene sutures (Ethicon, Inc., Somerville, New Jersey, USA).

### Surgical Techniques

Written informed consent was obtained from parents or guardians before surgery. Conduit size was determined by age and the body surface area and converted to z-score. A conduit with z-score over +1 was preferred up to 20 mm of the conduit diameter.

Standard cardiopulmonary bypass surgical procedures were performed. Intra-cardiac malformations were corrected. The end-to-end distal anastomosis was made away from the pulmonary artery bifurcation and leaving about 5 mm conduit above the valve. When the left pulmonary artery (LPA) and/or right pulmonary artery (RPA) were hypoplasia, extra bovine jugular vein patch was used for angioplasty before the implantation of DP-BJVC. Following the distal anastomosis, probes were used to measure the dimension of anastomosis and orifices of LPA and RPA. Next, the proximal orifice of DP-BJVC was anastomosed to the right ventricular infundibulum. All anastomoses were sutured continuously with single 6-0 or 5-0 Prolene suture.

Before discontinuation of cardiopulmonary bypass, transesophageal echocardiogram was performed to assess the hemodynamics of the neo-pulmonary arterial trunk. Before chest closure, the GORE® Pericardial Membrane (W.L. Gore and Associates, Arizona, USA) was used to keep the conduit and right atrium from adhering to the sternum in order to reduce the potential risks in any future sternotomy.

Postoperatively, antibiotics covering gram-negative and gram-positive bacteria were used for 3 days and then adjusted according to clinical assessment. The International Normalized Ratio was maintained between 1.6 and 2.0 with warfarin for 6 months.

### Follow-Ups

Follow-ups were made via clinic visits at the 1, 6, 12 months after operation and then annually, including physical examination, transthoracic echocardiography, and cardiac MRI or CT when appropriate. Last follow-up was completed between January to June, 2020. Echocardiography was performed to measure conduit diameter at the level of annulus and hemodynamics. The peak gradient across the conduit (PG) was the sum pressure gradients at the proximal, valvular, and distal levels. Conduit stenosis was defined as PG ≥50 mmHg (18). Conduit valve regurgitation was graded as none, trivial, mild, moderate and severe mainly according to the strength and size of the regurgitant jet (19). Patients with conduit stenosis or over moderate regurgitation received catheter based reintervention or surgical replacement.

The primary outcomes included patient death, conduit reintervention, and conduit explantation. Early mortality was defined as death within 30 postoperative days. The time from the date of the conduit implantation to the date of the conduit replacement or the last follow-up was defined as the time of freedom from conduit explantation. The time from the date of the conduit implantation to the date of first catheter-based reintervention or the last follow-up was defined as the time of freedom from reintervention.

Other demographic and clinical variables were collected, including gender, age, weight, height, diagnosis of CHD, diameter and z-score of the conduit, preoperative SpO_2_, previous cardiac corrective or palliative procedures, LPA and/or RPA angioplasty, cardiopulmonary bypass time and aortic cross clamp time.

### Statistical Analysis

Data were described as mean ± SD, median (range) or frequency (%) when appropriate. Early deaths were censored for analysis of freedom from conduit explantation and reintervention and the associated factors. The Kaplan-Meier method was used to estimate the survival, freedom from conduit explantation, and freedom from conduit first reintervention which was further compared between groups using Log-rank (Mantel-Cox) test. Stepwise forward Cox multivariable analyses were performed to assess the risk factors for mortality, conduit explantation, and conduit reintervention. Variables were retained when *P* < 0.10. Results were reported as corresponding hazard ratios (HR) with 95% confidence intervals (CI). The PG across the conduit in different follow-up time were compared using mixed-effects analysis with the Geisser-Greenhouse correction and Sidak's multiple comparisons test for *post-hoc* analysis. Spearman correlation coefficient was used to analyze the correlations between the change of conduit diameter (defined as the diameter of pulmonary artery measured at the latest echocardiography minus that at implantation) and hemodynamics. *P* < 0.05 was considered significant. Data analysis was performed using IBM SPSS Statistics 23.0 (SPSS Inc., Chicago, Illinois, USA) and GraphPad Prism 8.0 software (GraphPad Software, San Diego, California, USA).

## Results

### Demographics and Operative Data

Ninety patients (median age: 4.2 years, range: 0.2–20.0) were enrolled in this study, including 35 (38.9%) patients ≤ 3 years old. Detailed demographic data, diagnosis of CHD, previous cardiac operations and operative data of RVOT reconstruction using DP-BJVC are presented in Table 1. Fourteen (15.6%) patients underwent DP-BJVC implantation as part of palliative operation, including VSD closure and pulmonary artery reconstruction (classic repair) for patients with congenitally corrected transposition of the great arteries (*n* = 4), operation with Glenn shunt (*n* = 3) or without taking-down of previous Glenn shunt (*n* = 2), and operation without closure of ventricular septal defect (*n* = 5).

**Table 1:** Demographic and operative data of the cohort using DP-BJVC.

**Variables**	***n* = 90**
**Demographics**	
Age (years)	4.2 (0.2–20.0)
Gender (male: female)	48:42
Height (cm)	95.5 (55–172)
Weight (kg)	13.3 (3.5–51.0)
Body surface area (m^2^)	0.58 (0.23–1.56)
McGoon ratio	1.7 (1.0–3.3)
Preoperative SpO_2_ (%)	82 (34–98)
**Anatomic diagnoses**	
PA/VSD	41 (45.6%)
DORV/PS or DORV/PA	12 (13.3%)
AV diseases	11 (12.2%)
Truncus arteriosus	7 (7.8%)
TGA/PS or TGA/PA	6 (6.7%)
ccTGA/PS or ccTGA/PA	5 (5.6%)
PR with repaired TOF	5 (5.6%)
TOF/APV	2 (2.2%)
MV diseases	1 (1.1%)
**Previous cardiac operations**	
Systemic-to-pulmonary shunt	13 (14.4%)
TOF repair	5 (5.6%)
Bidirectional Glenn shunt	3 (3.3%)
SAV	2 (2.2%)
PDA stenting	2 (2.2%)
**Intraoperative characteristics**	
Cardiopulmonary bypass time (min)	173 (72–563)
Aortic cross-clamp time (min)	92 (0–239)
DP-BJVC diameter (mm)	16 (12–20)
DP-BJVC z-score	1.95 ± 0.11
Reinforced conduit	52 (57.8%)
**Major combined procedures**	
LPA angioplasty	16 (17.8%)
RPA angioplasty	6 (6.7%)
ncVSD management	8 (8.9%)
ECMO	5 (5.6%)

*AV, aortic valve; ccTGA, corrected transposition of the great arteries; DORV/PS, double-outlet right ventricle with pulmonary stenosis; ECMO, extracorporeal membrane oxygenation; LPA, left pulmonary artery; ncVSD, non-committed ventricular septal defect; PA/VSD, pulmonary atresia with ventricular septal defect; PR, pulmonary regurgitation; PDA, patent ductus arteriosus; RPA, right pulmonary artery; SAV, surgical aortic valvoplasty; TOF, tetralogy of Fallot; TOF/APV, TOF with absent pulmonary valve; TGA, transposition of the great arteries*.

**Figure 1:**
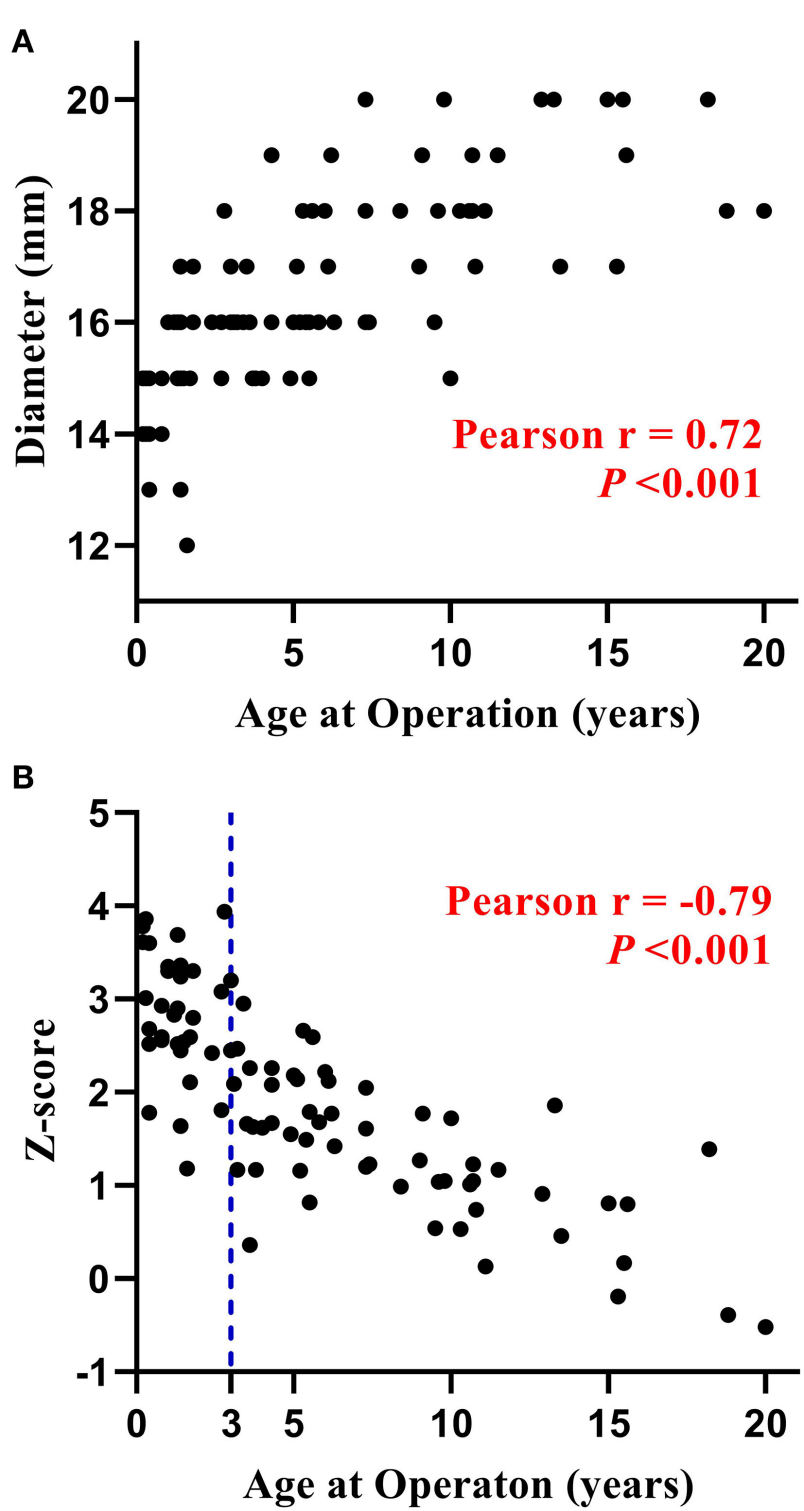
Distribution of the diameter **(A)** and z-score **(B)** of DP-BJVC at implantation. The conduit diameter (range from 12 to 20 mm) is significant positive corrected with patients' age (*P* < 0.001), while the conduit z-score is significant negative corrected with patients' age (*P* < 0.001). Particularly, four patients ≤ 3 years, as well as most of the older patients, received a conduit with z-score < +2.

The distribution of DP-BJVC size with age is shown in Figure 1A. The conduit diameter ranged from 12 to 20 mm (median 16), with mean z-score of +1.95. The conduit z-score trended to be lower with older age (Figure 1B). Specifically, four (11.4%) patients ≤ 3 years old and 42 (76.4%) aged 3 to 20 years received a conduit with z-score < +2.

### Follow-Up Results

Follow-up was completed in 83 (92%) patients. In the remaining seven (8%) patients, follow-up was lost at 1 (*n* = 3), 5 (*n* = 2), 6 (*n* = 1) and 8 (*n* = 1) years after DP-BJVC implantation. The duration of follow-up ranged 0.2 to 16.1 years (median 4.7).

#### Mortality

**Figure 2:**
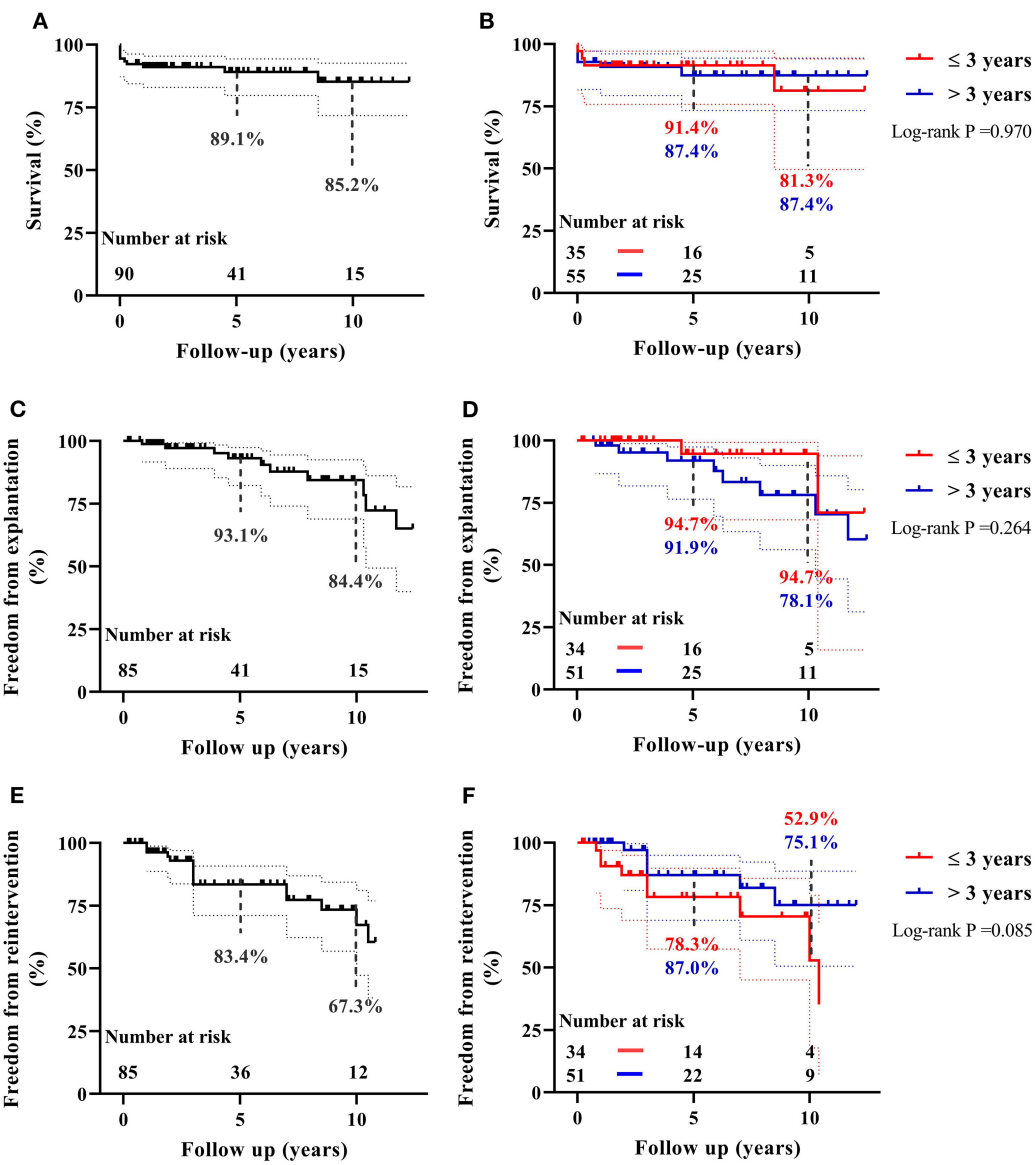
The Kaplan–Meier estimates of overall survival, freedom from DP-BJVC explantation, and freedom from first catheter-based reintervention for the entire cohort are shown in **(A)**, **(C)**, and **(E)**, respectively. The comparisons of these estimated values between children ≤ 3 years and older patients are shown in **(B)**, **(D)**, and **(F)**, respectively. Dashed lines represent 95% CIs. In summary, the performance of DP-BJVC in young children ( ≤ 3 years) is similar with that in larger patients.

There were five (5.6%) early deaths and five (5.6%) late deaths (Table 2) due to cardiac, pulmonary or cerebral events, and none from any DP-BJVC complications. One late death occurred on 65th days after operation while staying in the hospital, making the in-hospital mortality 6.7%. The Kaplan–Meier estimated overall survival was 89.1 and 85.2% at 5 and 10 years, respectively, for the entire cohort (Figure 2A). It was 91.4 and 81.3%, respectively, at 5 and 10 years in children ≤ 3 years old, similar to the older patients (87.4% both at 5 and 10 years) (*P* = 0.970, Figure 2B). Palliative procedure at the DP-BJVC implantation was the only risk factor for overall mortality both on univariate analysis (*P* = 0.028, Supplementary Tables 1, 2) and multivariate analysis (HR: 3.64, 95% CI: 1.03-12.93, *P* = 0.045).

**Table 2:** Operative data and the main causes for the early and late deaths (*n* = 10).

	**Age (years)**	**Diagnosis**	Surgical strategy^*^	**Conduit's hemodynamics**			**Survival time**	**Cause of death**
				Post-op. time^**^	**PG (mmHg)**	**PR**		
**Early deaths**								
Case 1	3.0	Truncus arteriosus	Rastelli	1 day	12	Trivial	2 days	Pulmonary hypertension crisis
Case 2	10.7	IE/AVS	Ross	1 day	8	None	9 days	Intracranial hemorrhage
Case 3	6.3	PA/VSD	Rastelli	1 day	10	None	11 days	LCOS
			ECMO					Multiple organs failure
Case 4	3.6	PA/VSD	RVOT reconstruction	10 day	26	Trivial	14 days	LCOS
			LPA angioplasty					
Case 5	7.3	AVS	Ross	20 days	6	None	24 days	Right heart failure
		Post- SAV	CABG (RCA)					LCOS
			Glenn shunt					
			ECMO					
**Late deaths**								
Case 6	1.4	DORV/PA	RVOT reconstruction	1 month	23	None	65 days	Sepsis
		Post-PDA stenting						Choking and asphyxia
								Intracranial hemorrhage after CPR
Case 7	0.3	PA/VSD	Rastelli	3 months	12	None	4 months	Choking and asphyxia
Case 8	10.3	ccTGA/PS	Senning	1 year	21	Trivial	13 months	Heart failure
			Rastelli					
Case 9	5.1	DORV/PS	Rastelli	4 years	8	Trivial	4.5 years	Sudden death (unknown reason)
Case 10	1.3	PA/VSD	RVOT reconstruction	7 years	18	Mild	8.5 years	Heart failure

*AVS, aortic valve stenosis; CPR, cardiopulmonary resuscitation; CABG, coronary artery bypass grafting; ccTGA/PS, corrected transposition of the great arteries with pulmonary stenosis; DORV/PA, double-outlet right ventricle with pulmonary atresia; DORV/PS, double-outlet right ventricle with pulmonary stenosis; ECMO, extracorporeal membrane oxygenation; IE, infective endocarditis; LPA, left pulmonary artery; LCOS, low cardiac output syndrome; PG, peak gradient (across the conduit); PR, pulmonary regurgitation; PA/VSD, pulmonary atresia with ventricular septal defect; PDA, patent ductus arteriosus; RVOT, right ventricular outflow tract; RCA, right coronary artery; SAV, surgical aortic valvoplasty; VSD, ventricular septal defect*.

* *Rastelli operation, including all Rastelli-type operations for patients with Truncus arteriosus, PA/VSD, and DORV/PS et al*.

** *Post-op. time, means the time from the date of operation to the date of last echocardiography examination*.

#### Explantation

Ten patients required DP-BJVC explantation at median of 6.1 years (range: 0.8 to 11.7) follow-up, including four (40%) with previous shunt operations (Table 3). The indications for explantation included conduit stenosis in five patients, among whom it was the distal anastomotic level in four and the middle of the conduit due to sternal compression in one; conduit aneurysmal dilatation with severe regurgitation in two patients and moderate regurgitation in 1, all being over 10 years after implantation without the annulus restraining device; infective endocarditis in two patients, with one at 2 months after catheter-based intervention resulting in severe regurgitation and the other with conduit valvular stenosis. Supplementary Figure 1 showed three examples of the failed DP-BJVCs with mild calcification and degeneration that was easily handled with tissue scissors.

**Table 3:** Operative data and the causes for explantations (*n* = 10).

	**Age (y)**	**Diagnosis**	**Surgical strategy**	**Conduit diameter (mm)**	**Conduit durability (years)**	**Causes of failure**	**Replacement material**
Case 1	15.0	PR	RVOT reconstruction	20	0.8	IE	Homograft
		Post TOF repair				PS (valvular)	
Case 2	9.8	AVS	Ross	20	1.8	PS (distal)	ePTFE conduit^*^
		Post SAV					
Case 3	9.5	Truncus arteriosus	Truncus repair	16	3.9	PS (distal)	DP-BJV patch^**^
						PR	
Case 4	2.7	PA/VSD	Rastelli	15	4.5	IE	ePTFE conduit
		Post B-T shunt				PR	
Case 5	9.1	DORV/PA/ncVSD Post B-T shunt	Rastelli (intra-ventricular conduit^***^)	19	5.9	PS (distal)	DP-BJVC
Case 6	13.5	PA/VSD	Rastelli	17	6.3	PS (distal)	DP-BJVC
		Post Melb. shunt				PR	
Case 7	5.2	TGA/PS/VSD	Rastelli	16	7.9	PS (middle)	DP-BJVC
			Glenn shunt				
Case 8	3.5	Truncus arteriosus	Truncus repair	17	10.3	PR	DP-BJVC
						Aneurysmal dilatation	
Case 9	0.4	TGA/PA/VSD	Rastelli	14	10.4	PR	DP-BJVC
		Post B-T shunt				Aneurysmal dilatation	
Case 10	3.2	TOF/APV	TOF repair	16	11.7	PR	DP-BJVC
						Aneurysmal dilatation	

*DORV/PA, double-outlet right ventricle with pulmonary atresia; ePTFE, expanded polytetrafluoroethylene; IE, infective endocarditis; ncVSD, non-committed ventricular septal defect; PR, pulmonary regurgitation; PS, pulmonary stenosis; PA/VSD, pulmonary atresia with ventricular septal defect; RVOT, right ventricular outflow tract; TGA/PA, transposition of the great arteries with pulmonary atresia; TOF/APV, tetralogy of Fallot with absent of pulmonary valve*.

* *ePTFE conduit, Gore-Tex vessel (W.L. Gore and Associates, Arizona, USA) equipped with hand-sewn 0.1 mm thick ePTFE valve*.

** *DP-BJV patch, valved decellularized-and-photooxidated bovine jugular vein patch*.

*** *Intra-ventricular conduit repair of DORV/ncVSD previous reported by our team (1)*.

The Kaplan-Meier estimated freedom from conduit explantation was 93.1 and 84.4% at 5 and 10 years, respectively, for the entire cohort (Figure 2C). It was 94.7% at 5 and 10 years in children ≤ 3 years old, similar to the older patients (91.9% at 5 years and 78.1% at 10 years) (*P* = 0.264, Figure 2D). On univariable analysis, lower preoperative SpO_2_ (*P* = 0.032) and previous cardiac operation (*P* = 0.018) were significantly associated with explantation (Supplementary Tables 1, 2). On multivariate analysis, previous cardiac operation was the only significant risk factor for conduit explantation(HR: 6.31, 95% CI: 1.12–35.66, *P* = 0.037).

#### Reintervention

Fifteen (16.7%) patients required 18 catheter-based reintervention during the follow-ups, including 16 (89%) balloon dilations, 7 for LPA, 6 for distal anastomosis, 2 for RPA, and 1 for conduit valve; and 2 (11%) stent placements in LPA.

The first reintervention occurs at median of 4.1 years (range: 0.8 to 10.5) follow-up. The Kaplan–Meier estimated freedom from first reintervention was 83.4 and 67.3% at 5 and 10 years respectively (Figure 2E). It was 78.3 and 52.9%, respectively, at 5 and 10 years in children ≤ 3 years old, which was not significantly different compared to older patients (87.0% at 5 years and 75.1% at 10 years) (*P* = 0.076, Figure 2F). On univariate analysis, PG before discharge (*P* = 0.008), previous cardiac operation (*P* = 0.091) and non-Ross operation (*P* = 0.084) were associated with increased reintervention rate (Supplementary Tables 1, 2). None of these were significant on multivariate analysis.

#### Conduit Hemodynamics

**Figure 3:**
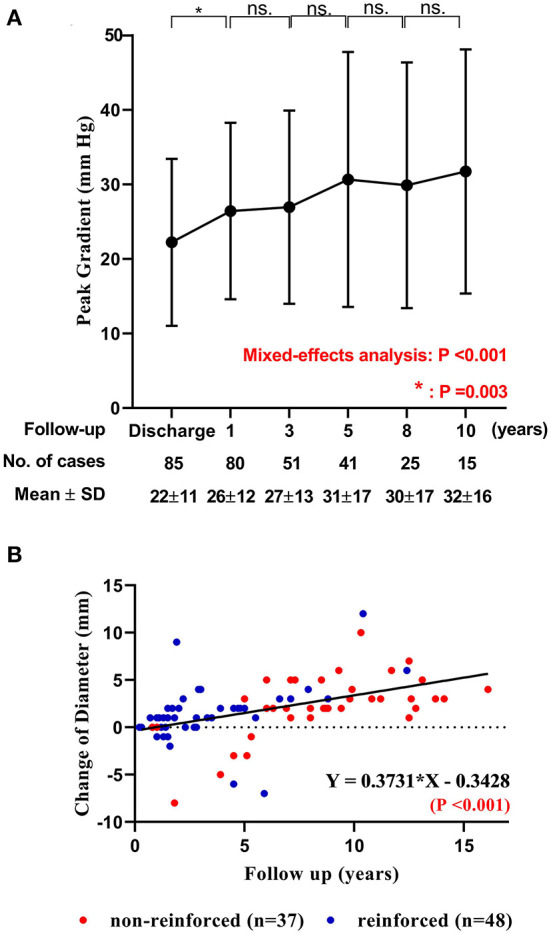
The change of peak gradient and diameter of DP-BJVC. **(A)** Peak pressure gradient across the conduit measured by echocardiography before discharge and at 1, 3, 5, 8 and 10 years after implantation are presented with mean and standard deviation (SD). The gradient significantly increased with time (*P* < 0.001). The comparisons of adjacent time show that the increase mainly occurred in the first postoperative year (*P* =0.003), which become slightly thereafter (*P* > 0.05). ns, no significance. **(B)** The change of DP-BJVC diameter measured by echocardiography at the annular level from implantation to the last follow-up significantly and positively linear correction with the follow-up years. Each dot represents one patient. The diameter change of reinforced conduits (red dots) have similar regression coefficient with the non-reinforced conduits (blue dots. *P* = 0.806). Four conduits (arrows) had a diameter decrease over 5 mm early after implantation, which were all caused by stenosis and had been replaced.

Before hospital discharge after the initial implantation, the median PG across the conduit was 23.0 mmHg (range: 2.0 to 40.0 mmHg). It became significantly higher during follow-ups (*P* < 0.001). The annual increase in PG was highest in the first postoperative year (*P* = 0.003), but without any significant annual difference in the subsequent consecutive year-to-year comparisons (Ps > 0.30, Figure 3A and Supplementary Figure 2).

Eleven (12.9%) patients had conduit stenosis during follow-up with median PG of 68.0 mmHg (ranging 52.0 to 92.0), including 8 at the distal anastomosis level, 2 at the valvular level, and 1 at the middle of the conduit. Six of them were alleviated by balloon dilation, and 5 ultimately received conduit explantation as described above.

Before hospital discharge, conduit valve regurgitation was described as trivial in 27 patients and none in the remaining. Conduit regurgitation measured at the last echocardiography was graded as trivial in 48 patients, mild in 6, moderate in 3, severe in 2, and none in the remaining. Both the two patients with severe regurgitation received conduit explantation as described above.

No serious thrombotic events were found during follow-up. Supplementary Figures 3, 4 provided the follow-up echocardiography, CT, and MRI examples for DP-BJVC, and the example of aneurysmal dilatation.

#### Change in Conduit Diameter

The overall change of conduit diameter significantly and positively correlated with follow-up time (*P* < 0.001), with regression coefficient of 0.37 (Figure 3B). The change of conduit diameter did not significantly correlate with the change of PG (defined as the PG measured at the last echocardiography minus that before discharge. *P* = 0.940), or the change of regurgitation grade (defined as the grade of regurgitation measured at the last echocardiography minus that before discharge. *P* = 0.680). The diameter change of reinforced conduit has similar regression coefficient with the non-reinforced conduit (*P* = 0.806. Supplementary Figure 5). Of note, four conduits had a diameter decrease over 5 mm after implantation (marked by arrows in Figure 3B) due to stenosis mainly at the distal anastomosis, which had been replaced (case 2 to 5 in Table 3).

## Discussion

We examined the midterm outcomes of our novel designed DP-BJVC for RVOT reconstruction in children with CHD (Graphical Abstract). The overall survival rate was 85.2% at 10 years. The 10-year freedom from conduit explantation and reintervention was 84.4 and 67.3%, respectively. These figures were largely comparable between children ≤ 3 years old and the older patients.

The early mortality in our cohort was 5.6%, which was higher compared to previous reports up to 4.1% (3, 4, 20). This reflects the developing course in our center. Two of them received the Ross operation that was being implemented as learning curve. The other three deaths were due to the much older age at operation resulting in poorer cardiac and pulmonary vascular function. Indeed, the age at operation in children with CHD is generally older in our cohort (Table 1), representing the current situation in China and other developing countries. Nonetheless, midterm survival of our cohort is comparable to previous reports on RVOT reconstruction in children with CHD (3, 4, 9, 20). The significant risk factor for overall mortality was palliative procedures, which are usually associated with more complex structural deformities and un-physiological postoperative hemodynamics.

The notion for us to design DP-BJVC included the followings: (1) It is a cell-free xenogeneic scaffold, thus preventing the immunogenicity from donor cells (21); (2) It is a non-glutaraldehyde-treated BJVC, thus avoiding the toxic effects of glutaraldehyde (11); (3) the structure and composition of the extracellular matrix are preserved by photooxidation and its effective crosslink with the collagen proteins (22). The hypothesized advantages were demonstrated in a series of experimental studies *in vitro* and *in vivo* by our team (15–17, 23, 24). The tissue structure of DP-BJVC was stable after 12-week subcutaneous implantation in rats, with all-layer host cells repopulation and neo-capillaries formation (16). The conduit showed good valvular function without calcification after 6-month implantation in dogs, with the host cells infiltrated and migrated from outer layer to the middle layer of the conduits' walls (17).

These advantages are further demonstrated in the present cohort. The midterm durability of DP-BJVC is satisfactory and comparable with that of glutaraldehyde-treated BJVC, in which the 10-year freedom rate from replacement ranged from 59 to 90% (4, 7, 20, 25). Previous cardiac operation was the only risk factor for conduit explantation as most of those procedures were for pulmonary vascular dysplasia, which limits the size of distal anastomosis with heavier afterload for the conduit. More importantly, DP-BJVC appeared to do equally well in younger children, that is, ≤ 3 years old, which is, in other words, better than previously reported cohorts. Younger age has been identified as the independent risk factor for conduit failure (2–4). The Congenital Heart Surgeons Society in 2006 year reported that 58% of children ≤ 2 years old required reintervention within 3 years of implantation using glutaraldehyde-treated BJVC for RVOT reconstruction (26). Another single-center reports by Ugaki et al. demonstrated the 10-years freedom from BJVC replacement rate of 37.1%, with age <3 years as an independent risk factor (27).

Several factors may be attributable to the good durability of DP-BJVC for younger children. Firstly, the conduits used for young children were greater in z-score than that for older patients (Figure 1B). Secondly, DP-BJVC showed an increase in diameter with age, that is, conduit dilatation probably resulted from calcification resistance as non-glutaraldehyde treated (24). We found that calcification in the failed DP-BJVC was mild and could be easily handled. The degree of dilatation is further refined by the flexible Dacron restraining device. All the 3 conduits developed aneurysmal dilatation and valvular regurgitation were implanted before 2013 without such device.

The PG across the DP-BJVC increased over time although only reaching statistical significance in the first year. This may be attributable to fairly fast somatic growth in young children. Additionally, the short-term anticoagulant therapy postoperatively might be helpful to reduce the subclinical conduit thrombosis (18). BJVC-related distal anastomotic stenosis has been reported as one of the main reasons for conduit failure. There were eight (9.4%) patients with distal anastomotic stenosis (PG ranged ≥50 mmHg) during the entire follow-up of our study, which trended to be less than that using glutaraldehyde-treated BJVC, with reported incidence range 13.5 to 22.0% (3, 7, 18). This finding may be as a result of better biocompatibility with lower inherent immunogenicity (16, 28). In contrast, the glutaraldehyde-treatment has been reported as a contributor to the formation of neo-intimal peeling at the distal anastomosis (18, 29).

Late infective endocarditis is a particular concern for BJVC (25). In a systematic review involving 840 patients from 55 studies using Contegra conduits, the pooled incidences of endocarditis was 7.1% during follow-up period ranging 9 to106 months (30). Furthermore, the infection usually occurred late after BJVC implantation, and its risk increased with time (31). In our cohort, only two (2.4%) patients developed with infective endocarditis during follow-up. This may be attributable to the favorable hemodynamics and the lack of thrombosis as the site for bacterial adhesion due to anticoagulation treatment (1, 32).

## Limitations

Our study has a couple of limitations. First, this is a cohort study, rather than a randomized and control trial. There were no control group using other types of conduits. During the study period, DP-BJVC was the only available material for RVOT reconstruction in our center, except for cryopreserved homograft used in a few children and ePTFE valved conduit (14) used in adolescents and adults recently. Second, the indications for conduit reintervention remain controversial and have been broadened over the past decade, which has influenced our management. The incidence of conduit reintervention substantially increased in the past 5 years.

## Conclusion

Our novel designed DP-BJVC performed satisfactory functionality and durability in midterm follow-up, particularly for those younger than or equal to 3 years old. It showed advantages in resistance to calcification and infection, as well as appropriate dilation with age. DP-BJVC is a promising alternative for RVOT reconstruction in children with CHD.

## Data Availability Statement

The raw data supporting the conclusions of this article will be made available by the authors, without undue reservation.

## Ethics Statement

The clinical application of the conduit was approved by the IEC in April 1, 2004 (reference number: LYF2004011). The study protocol was approved in January 10, 2020 (reference number: LYF2020097). Written informed consent to participate in this study was provided by the participants' legal guardian/next of kin.

## Author Contributions

J-GH and Z-SW developed the conduit. Z-SW and JL make substantial contributions to conception and design. TQ contributed to data collection and draft the article. All authors contributed to the article and approved the submitted version.

## Conflict of Interest

The authors declare that the research was conducted in the absence of any commercial or financial relationships that could be construed as a potential conflict of interest.

## Publisher's Note

All claims expressed in this article are solely those of the authors and do not necessarily represent those of their affiliated organizations, or those of the publisher, the editors and the reviewers. Any product that may be evaluated in this article, or claim that may be made by its manufacturer, is not guaranteed or endorsed by the publisher.

## Supplementary Material

Supplementary Figure 1 Intraoperative photographs of failed DP-BJVC in two patients. **(A)** 10.4 years after implantation in Rastelli operation for pulmonary atresia, the lumen surface of the conduit was smooth without visible thrombosis and calcification. **(B)** 10.3 years after implantation for truncus arteriosus repair, the conduit was aneurysmal dilated with mild calcification. Residual valve tissue is displayed with tweezers.

Supplementary Figure 2 Results of mixed-effects analysis and Sidak's multiple comparisons of peak gradient across the DP-BJVC during follow-ups. Peak gradient across the conduit significant increased during follow-up (*P* = 0.0019). The difference mainly occurred during the first year after implantation (t1 vs. t0, *P* = 0.0027), and no significant difference was found thereafter (Ps > 0.3). t0, before discharge (*n* = 85); t1, 1 year after implantation (*n* = 80); t2, 3 years after implantation (*n* = 51); t3, 5 years after implantation (*n* = 41); t4, 8 years after implantation (*n* = 25); t5, 10 years after implantation (*n* = 15).

Supplementary Figure 3 Examples of imaging examinations of implanted DP-BJVC. **(A,B)** Echocardiography at 2 years after Rastelli operation for pulmonary atresia. The conduit valve leaflets (PV) are clearly visible in 2-dimensional in the long-axis view along the conduit. Pulse-wave Doppler signal at conduit level showed normal laminar flow. **(C,D)** Cardiac CT examination at 10 years after truncus arteriosus repair. The conduit (PA) was mildly dilated in the proximal end, with scattered calcification at the distal (white arrow). The size of the right ventricle was normal. **(E,F)** Cardiac CT examination at 11 years after Rastelli operation for pulmonary atresia. The conduit was well-shaped with scattered calcification. **(G,H)** Cardiac MRI at 12 years after Rastelli operation for corrected transposition of the great arteries, showing the normal-sized heart and unobstructed ventricular outflow tracts.

Supplementary Figure 4 Severe aneurysmal dilatation of DP-BJVC in a 3.5 years old girl. The conduit was reinforced with a Dacron ring. However, the diameter dilated almost 170% (from 12 to 20 mm) after 23 months at the level of annulus, which was even larger at the proximal and distal level. Although the conduit is currently in a undesirable state due to the extremely heavy afterload, it indirectly reflect that the annulus restraining device does not strictly limit the diameter of the conduit.

Supplementary Figure 5 The results of comparison of diameter change between reinforced and non-reinforced DP-BJVC. The change of diameter significantly and positively linear correlated with follow-up time both in non-reinforced and reinforced conduit, with regression coefficient (slopes) of 0.53 and 0.48 respectively. The differences between the slopes are not significant (*P* = 0.806).
