# Supplementary figures and images for: Midterm Outcomes of Crosslinked Acellular Bovine Jugular Vein Conduit for Right Ventricular Outflow Tract Reconstruction
Source: Front Pediatr. 2021 Aug 17;9:725030. doi: 10.3389/fped.2021.725030 (PMC8416030; doi:10.3389/fped.2021.725030)

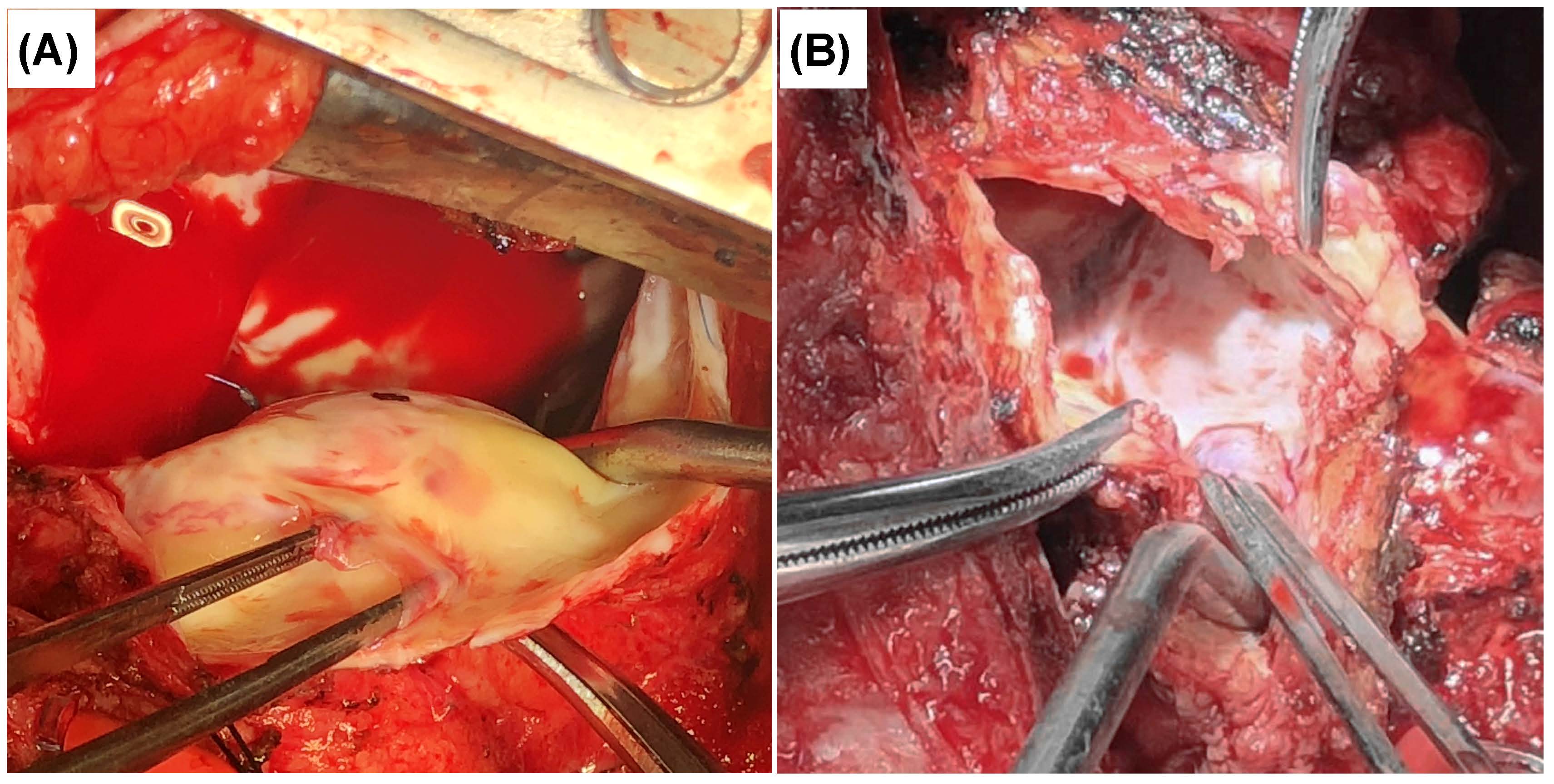

Supplement: Supplementary Figure 1 — Intraoperative photographs of failed DP-BJVC in two patients. (A) 10.4 years after implantation in Rastelli operation for pulmonary atresia, the lumen surface of the conduit was smooth without visible thrombosis and calcification. (B) 10.3 years after implantation for truncus arteriosus repair, the conduit was aneurysmal dilated with mild calcification. Residual valve tissue is displayed with tweezers. [file Image_1.JPEG]

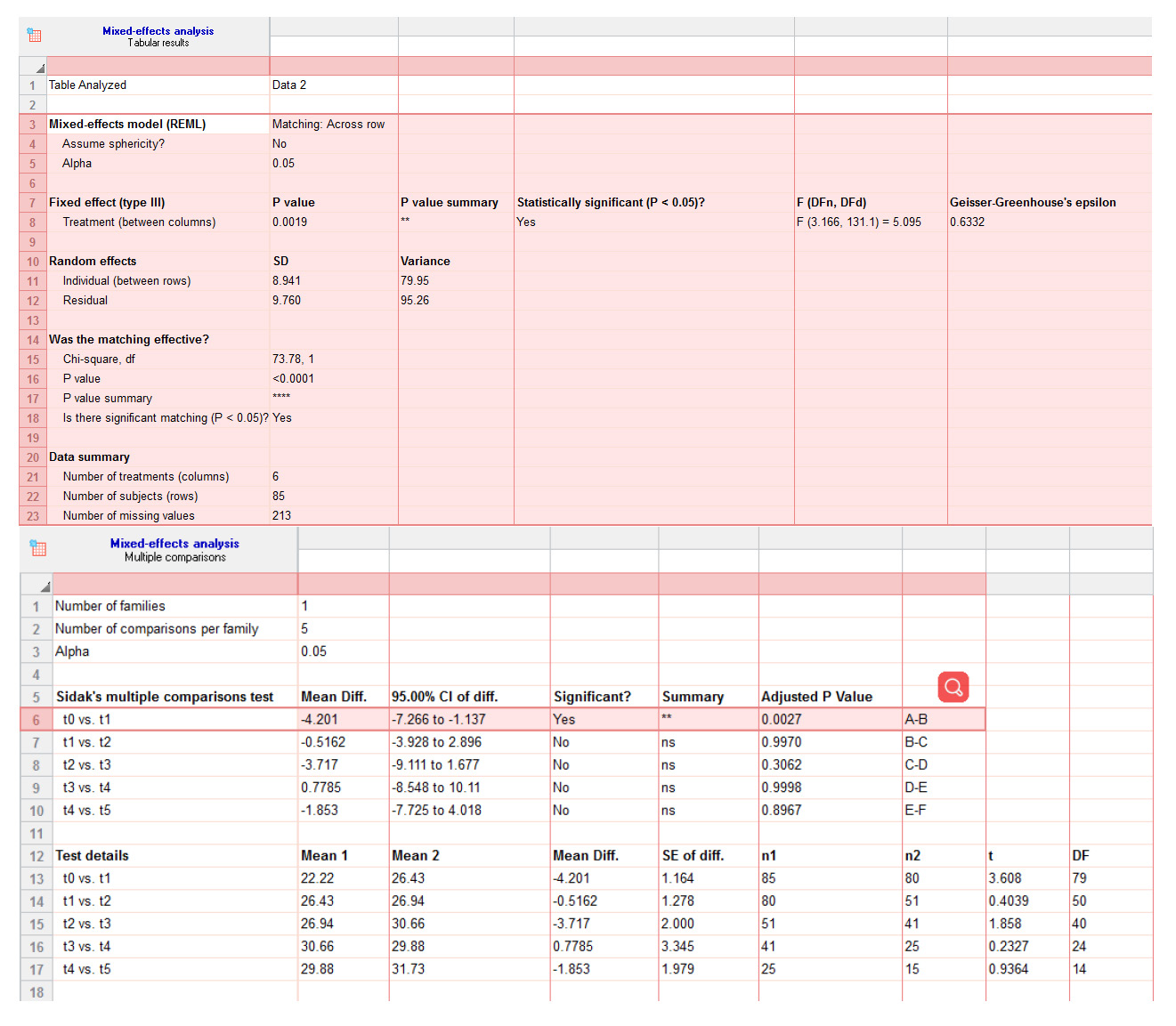

Supplement: Supplementary Figure 2 — Results of mixed-effects analysis and Sidak's multiple comparisons of peak gradient across the DP-BJVC during follow-ups. Peak gradient across the conduit significant increased during follow-up (P = 0.0019). The difference mainly occurred during the first year after implantation (t1 vs. t0, P = 0.0027), and no significant difference was found thereafter (Ps > 0.3). t0, before discharge (n = 85); t1, 1 year after implantation (n = 80); t2, 3 years after implantation (n = 51); t3, 5 years after implantation (n = 41); t4, 8 years after implantation (n = 25); t5, 10 years after implantation (n = 15). [file Image_2.JPEG]

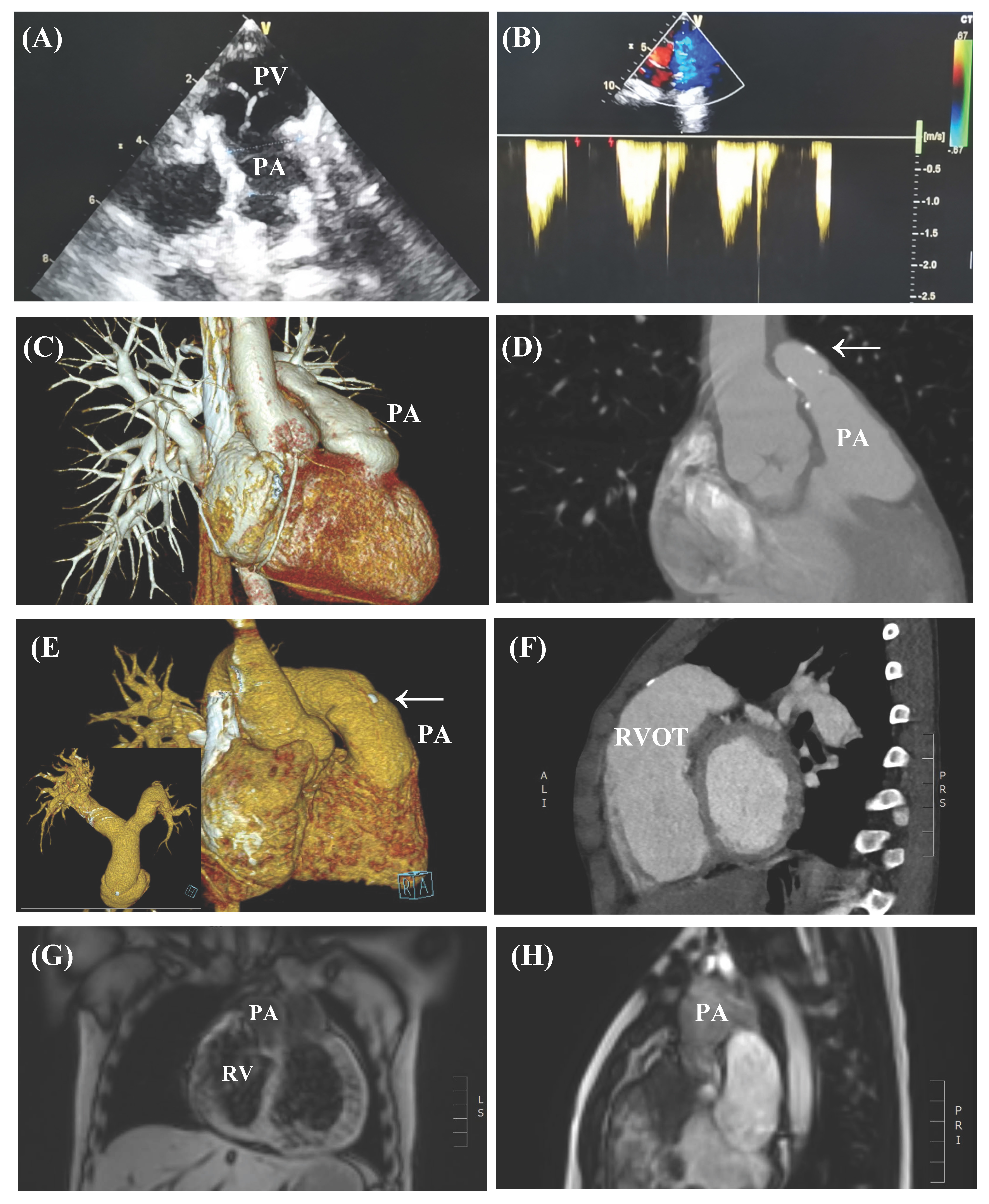

Supplement: Supplementary Figure 3 — Examples of imaging examinations of implanted DP-BJVC. (A,B) Echocardiography at 2 years after Rastelli operation for pulmonary atresia. The conduit valve leaflets (PV) are clearly visible in 2-dimensional in the long-axis view along the conduit. Pulse-wave Doppler signal at conduit level showed normal laminar flow. (C,D) Cardiac CT examination at 10 years after truncus arteriosus repair. The conduit (PA) was mildly dilated in the proximal end, with scattered calcification at the distal (white arrow). The size of the right ventricle was normal. (E,F) Cardiac CT examination at 11 years after Rastelli operation for pulmonary atresia. The conduit was well-shaped with scattered calcification. (G,H) Cardiac MRI at 12 years after Rastelli operation for corrected transposition of the great arteries, showing the normal-sized heart and unobstructed ventricular outflow tracts. [file Image_3.JPEG]

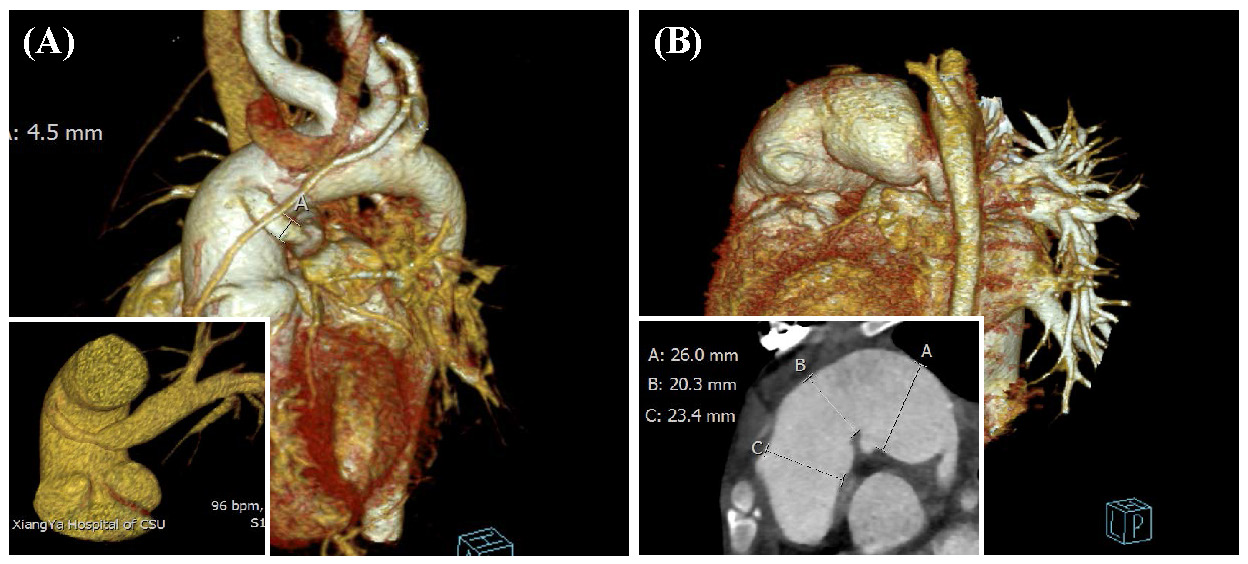

Supplement: Supplementary Figure 4 — Severe aneurysmal dilatation of DP-BJVC in a 3.5 years old girl. The conduit was reinforced with a Dacron ring. However, the diameter dilated almost 170% (from 12 to 20 mm) after 23 months at the level of annulus, which was even larger at the proximal and distal level. Although the conduit is currently in a undesirable state due to the extremely heavy afterload, it indirectly reflect that the annulus restraining device does not strictly limit the diameter of the conduit. [file Image_4.JPEG]

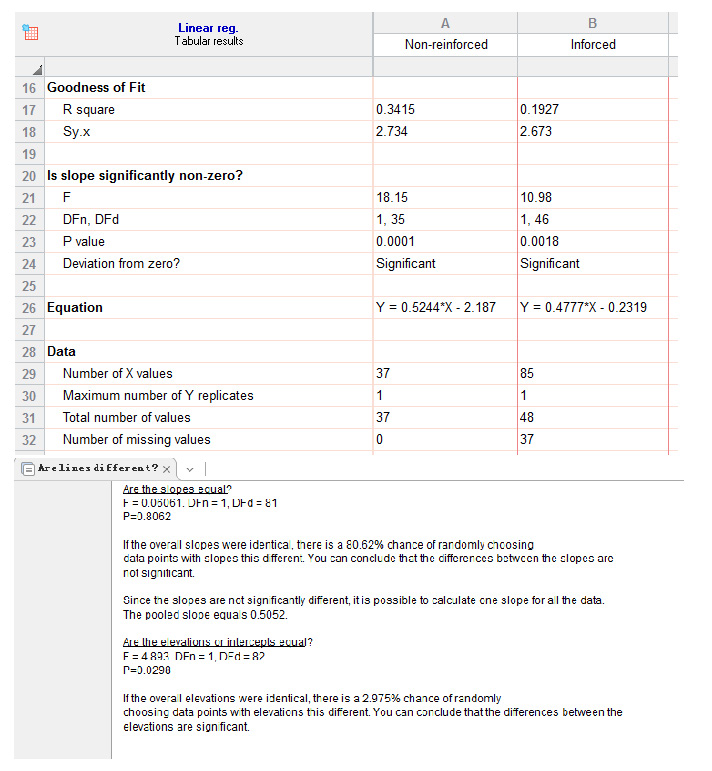

Supplement: Supplementary Figure 5 — The results of comparison of diameter change between reinforced and non-reinforced DP-BJVC. The change of diameter significantly and positively linear correlated with follow-up time both in non-reinforced and reinforced conduit, with regression coefficient (slopes) of 0.53 and 0.48 respectively. The differences between the slopes are not significant (P = 0.806). [file Image_5.JPEG]
