# Supplementary material for: Midterm Outcomes of Crosslinked Acellular Bovine Jugular Vein Conduit for Right Ventricular Outflow Tract Reconstruction
Source: Front Pediatr. 2021 Aug 17;9:725030. doi: 10.3389/fped.2021.725030 (PMC8416030; doi:10.3389/fped.2021.725030)
Supplement: Supplementary file 6 [file Data_Sheet_1.docx]

**Suppl. Table 1. Factors association with adverse outcomes analyzed on univariate Cox regression in 90 patients undergoing RVOT reconstruction using DP-BJVC**

| Variables | *P* value | | |
| --- | --- | --- | --- |
|  | Overall death | Replacement | Reintervention |
| Continuous variables |  |  |  |
| Age | 0.578 | 0.545 | 0.582 |
| Weight | 0.720 | 0.243 | 0.959 |
| Height | 0.608 | 0.144 | 0.662 |
| BSA | 0.638 | 0.186 | 0.883 |
| Preoperative SpO_2_ | 0.783 | 0.032 | 0.817 |
| Preoperative McGoon index | 0.790 | 0.428 | 0.275 |
| Diameter of DP-BJVC | 0.776 | 0.138 | 0.444 |
| Z-score of DP-BJVC | 0.545 | 0.469 | 0.402 |
| Binary variables |  |  |  |
| Sex (male vs. female) | 0.545 | 0.166 | 0.407 |
| Previous cardiac operation (Y vs. N) | 0.738 | 0.018 | 0.091 |
| Restrained conduit (Y vs. N) | 0.639 | 0.322 | 0.218 |
| Ross operation (Y vs. N) | 0.328 | 0.106 | 0.084 |
| Non-anatomic repair (Y vs. N) | 0.028 | 0.523 | 0.115 |
| LPA/RPA angioplasty (Y vs. N) | 0.292 | 0.379 | 0.426 |

BSA, body surface area; PG, peak pressure gradient across the conduit; Y vs. N, Yes versus No; LPA/RPA, left or right pulmonary artery.

**Suppl. Table 2.** Diagnosis of multi-collinearity by multiple linear regression on variables included in Cox analysis

| Model | | Unstandardized Coefficients | | Standardized Coefficients | t | Sig. | Collinearity Statistics | |
| --- | --- | --- | --- | --- | --- | --- | --- | --- |
|  |  | B | Std. Error | Beta |  |  | Tolerance | VIF |
|  | (Constant) | .572 | 1.460 |  | .392 | .696 |  |  |
|  | Age | .023 | .048 | .193 | .485 | .629 | .075 | 13.360 |
|  | Height | .007 | .051 | .359 | .143 | .887 | .002 | 530.420 |
|  | Weight | -.014 | .205 | -.252 | -.066 | .947 | .001 | 1231.305 |
|  | BSA | .112 | 12.020 | .060 | .009 | .993 | .000 | 3442.769 |
|  | Conduit diameter | -.054 | .156 | -.166 | -.345 | .731 | .051 | 19.650 |
|  | Conduit z-score | .168 | .240 | .293 | .701 | .486 | .068 | 14.809 |
|  | SpO2 | .002 | .007 | .051 | .352 | .726 | .559 | 1.789 |
|  | McGoon | -.167 | .188 | -.109 | -.888 | .377 | .778 | 1.285 |
|  | Sex | -.197 | .140 | -.166 | -1.409 | .163 | .854 | 1.172 |
|  | Ross operation | -.137 | .244 | -.079 | -.562 | .576 | .605 | 1.652 |
|  | Previous cardiac operation | -.253 | .163 | -.192 | -1.557 | .124 | .781 | 1.280 |
|  | LPA/RPA angioplasty | -.031 | .166 | -.023 | -.187 | .852 | .812 | 1.231 |
|  | Non-anatomic repair | .178 | .189 | .109 | .941 | .350 | .882 | 1.134 |

Patients’ age, height, weight, BSA, the diameter and z-score of the conduits may have multicollinearity (VIF >10). The remaining variables (including preoperative SpO_2_ and previous cardiac operation) are thought to be independent.

VIF, variance inflation factor.
